# Acute Food Protein-Induced Enterocolitis Syndrome Selectively Induced by Durum Wheat: A Case Report

**DOI:** 10.7759/cureus.106401

**Published:** 2026-04-03

**Authors:** Go Kusakawa, Yuki Murata, Naoki Kajita, Kumiko Morita, Kouichi Yosida

**Affiliations:** 1 Division of Allergy, Tokyo Metropolitan Children's Medical Center, Fuchu, JPN; 2 Department of Pediatrics, Japanese Red Cross Medical Center, Shibuya, JPN; 3 Division of Allergy, Tokyo Metropolitan Children’s Medical Center, Fuchu, JPN

**Keywords:** durum wheat, food protein-induced enterocolitis syndrome, fpies, non-ige-mediated food allergy, oral food challenge, pediatric food allergy, wheat allergy

## Abstract

Food protein-induced enterocolitis syndrome (FPIES) can be triggered by wheat; however, whether clinical reactivity differs among wheat species remains unclear. We report a child who developed reproducible delayed vomiting after ingestion of pasta made from durum wheat at one year of age, while tolerating comparable amounts of other common wheat-based foods such as bread and noodles. An oral food challenge (OFC) confirmed the diagnosis despite incomplete fulfillment of consensus criteria. The patient subsequently achieved tolerance, as demonstrated by a negative follow-up OFC. This case suggests that wheat species-specific differences in antigenicity may influence clinical reactivity in FPIES. When clinical history raises the possibility that reactions differ according to specific wheat species, clinicians should not hesitate to perform species-specific OFCs with the relevant wheat sources. Such an approach is important to establish an accurate diagnosis and to avoid unnecessary dietary elimination. In addition, OFC is important not only for establishing the diagnosis but also for confirming tolerance acquisition.

## Introduction

Food protein-induced enterocolitis syndrome (FPIES) is a non-IgE-mediated food allergy characterized by delayed gastrointestinal symptoms, typically occurring one to four hours after ingestion of the causative food, including repetitive vomiting, lethargy, pallor, and sometimes diarrhea. In severe cases, hypotension and hypothermia may occur, requiring urgent medical care. Wheat is one of the commonly recognized triggers [1-3]. However, wheat is generally treated as a single antigen source, and little is known about whether clinical reactivity differs among wheat species. Wheat comprises multiple species with distinct protein compositions, including common wheat (*Triticum aestivum*) and durum wheat (*Triticum turgidum subsp. durum*), which differ in gluten structure and protein content [4]. Despite these differences, the potential impact of species-specific antigenicity on FPIES has not been clarified, and the role of specific food antigens in FPIES remains incompletely understood [5].

This issue is clinically important because wheat is widely consumed and represents an important component of the daily diet, including in Japan, where it is commonly consumed alongside rice. Uniform dietary elimination may impose a substantial burden on patients and their families. If reactivity differs according to wheat species, more tailored evaluation may help avoid unnecessary dietary restrictions.

Another important consideration is that the diagnosis of FPIES can be challenging due to its nonspecific presentation and often leads to delayed recognition [6]. Although adjunctive diagnostic approaches have been explored, including for wheat-induced FPIES [7], oral food challenge (OFC) remains essential for establishing the diagnosis and guiding management [8,9].

Against this background, we report a pediatric case of acute FPIES triggered by durum wheat in a patient who tolerated common wheat products. This case highlights the potential role of wheat species-specific antigenicity and the clinical value of targeted OFC in both diagnosis and dietary management.

## Case presentation

A 3-year-old male patient started weaning at the age of 6 months and was able to eat bread and udon (Japanese wheat noodles) without symptoms, consuming approximately 1 slice of bread (30-40 g; approximately 3,000-4,000 mg of protein) or a standard serving of udon noodles (100-150 g cooked weight; approximately 3,000-5,000 mg of protein) per meal, and thereafter consumed bread almost daily. At the age of 1 year, he ingested pasta (approximately one full, age-appropriate serving, corresponding to 50-80 g cooked weight; approximately 2,000-3,000 mg of protein) for the first time and developed repetitive vomiting approximately two hours after ingestion, without lethargy, pallor, diarrhea, hypotension, or other systemic symptoms. No cutaneous or respiratory symptoms suggestive of IgE-mediated allergy were observed. In addition, the patient had no fever or other signs of infection, and no family members had similar symptoms. He remained in good general condition. Medical attention was not sought. A few days later, he again ingested a similar age-appropriate serving of pasta (approximately 50-80 g cooked weight; approximately 2,000-3,000 mg of protein) and experienced repetitive vomiting approximately two hours later, demonstrating reproducibility of symptoms. At the age of three years, he ingested pasta and experienced repetitive vomiting two hours later, as he had at the age of 1 year. He never ingested pasta again without vomiting.

He was born at full term with a normal birth weight. He had no growth or developmental delays. He experienced repetitive vomiting within two hours of ingesting cow’s milk formula at the age of three months, prompting his physician to suspect cow’s milk-induced FPIES. Because the coronavirus disease 2019 pandemic was at its height at the time, the patient was given a hypoallergenic formula but received no close examination. By the age of two years, he was able to ingest cow’s milk without experiencing allergic symptoms. Atopic dermatitis was diagnosed at the age of one year but remitted by the age of three years. His parents had seasonal allergic rhinitis but no history of any other allergic disease.

At the age of 3 years, the patient had total IgE 1,600 IU/mL and specific IgE antibodies for wheat, ω-5-gliadin, and gluten at 0.71 kUA/L, <0.10 kUA/L, and <0.10 kUA/L, respectively (ImmunoCAP, Thermo Fisher Scientific, Uppsala, Sweden). A skin prick test using allergen extracts (Torii Pharmaceutical Co., Ltd., Tokyo, Japan) was negative for wheat. In addition, skin prick-to-prick tests with durum wheat and common wheat were also negative.

Based on these findings, acute FPIES due to durum wheat was suspected, although the patient did not fulfill the diagnostic criteria of the consensus guidelines [8]. After written informed consent was obtained from his guardian to perform tests for a definitive diagnosis, an OFC with 5 g of pasta was performed (NIPPN Co., Ltd., Tokyo, Japan; approximately 250 mg of protein). The OFC was conducted as a day-hospital procedure under the supervision of physicians and nurses, with continuous monitoring and in the presence of the guardian. A single dose of 5 g of pasta was administered, and the patient was observed for the development of symptoms. The patient vomited twice approximately two hours after ingestion, without lethargy, pallor, diarrhea, hypotension, hypothermia, or other systemic symptoms. No cutaneous or respiratory symptoms were observed, and the patient remained in good general condition without signs of infection. Although his symptoms did not fully meet the OFC diagnostic criteria defined in the 2017 international consensus guidelines for FPIES [8], the reproducibility of delayed vomiting following isolated ingestion of durum wheat strongly supported the diagnosis [6]. One year after the diagnostic OFC, a repeat OFC with pasta made from durum wheat was performed after written informed consent was obtained. The patient tolerated the challenge without vomiting or any other gastrointestinal, systemic, skin, or respiratory symptoms. Following confirmation of a negative OFC, dietary counseling was provided, and gradual reintroduction of durum wheat at home was initiated. The patient subsequently consumed durum wheat-containing foods in age-appropriate daily amounts comparable to those of common wheat products (e.g., approximately 50-80 g of cooked pasta per meal; approximately 2,000-3,000 mg of protein) without recurrence of symptoms, confirming the acquisition of tolerance.

**Figure 1:**
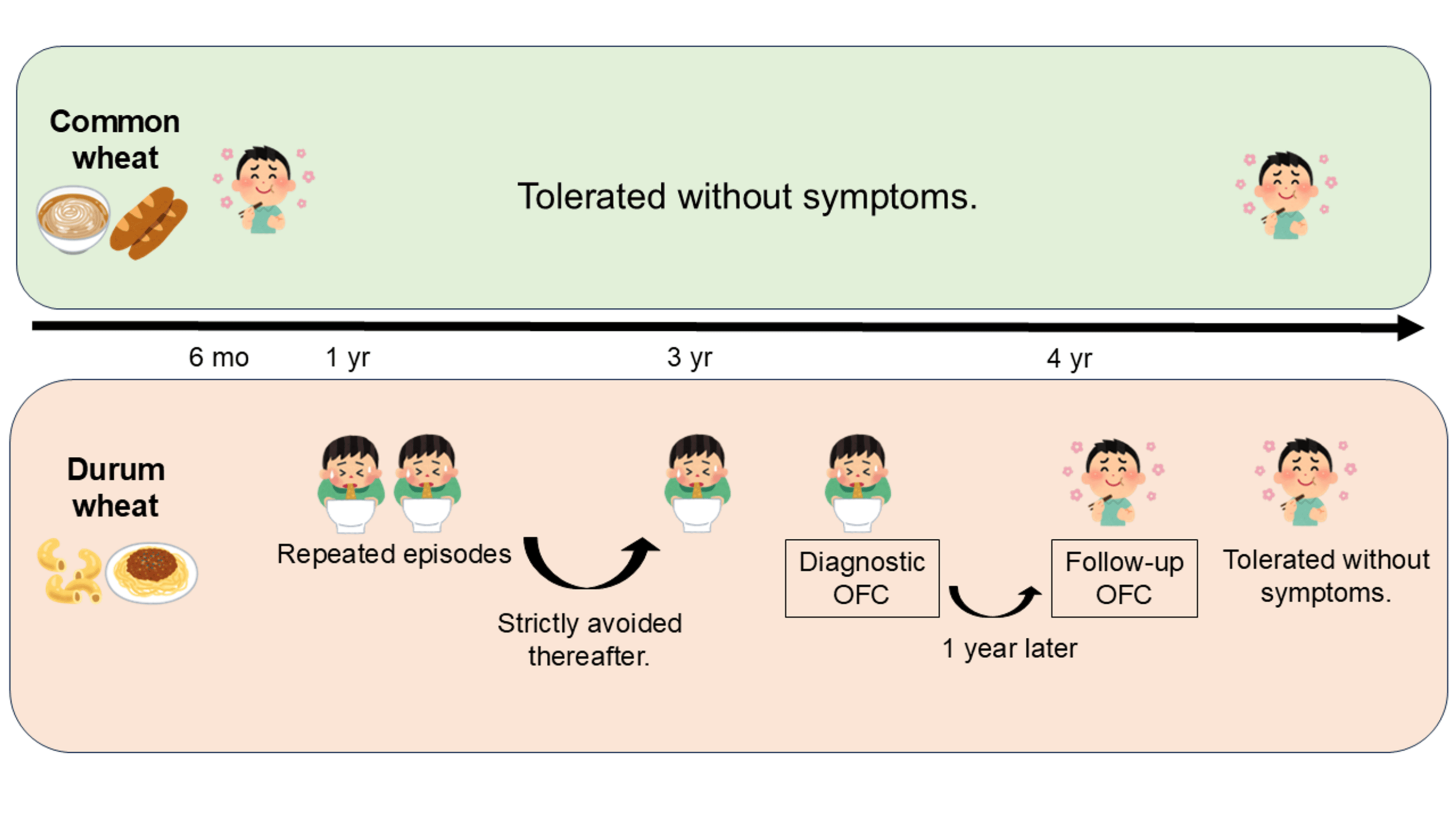
Clinical course of FPIES selectively induced by durum wheat Clinical course of a patient with FPIES selectively induced by durum wheat. The patient tolerated common wheat but developed repetitive vomiting after ingestion of durum wheat. Diagnosis and tolerance acquisition were confirmed by oral food challenge (OFC). The figure was created using Microsoft PowerPoint (Microsoft Corporation, Redmond, WA, USA). FPIES: food protein-induced enterocolitis syndrome

The clinical course of the patient is summarized in Figure 1.

As a result, all dietary restrictions related to wheat products were lifted, and no limitations in daily life have been required.

## Discussion

In IgE-mediated wheat allergy, antigenic differences among wheat species have garnered little attention, and the Japanese guidelines for food allergy group udon noodles and bread (made from common wheat) into the same food category for the purposes of OFC. However, the present case raises the possibility that species-specific differences may exist in FPIES. Therefore, when clinical history suggests reproducible symptoms associated with a particular wheat product, evaluating reactivity at the level of specific wheat species using targeted OFC may help avoid unnecessary dietary elimination.

Although the specific cause of symptoms induced only by durum wheat in this case is unclear, durum wheat is known to differ from common wheat in protein composition, including gluten structure and glutenin content, and may have relatively higher protein content depending on the preparation [4]. However, differences in protein quantity alone are unlikely to explain the present findings. The patient tolerated comparable or greater amounts of common wheat products without symptoms, further supporting that factors other than protein quantity were involved. Instead, differences in wheat composition, including antigenic differences between wheat species, may have contributed. However, as this is a single case without supporting prior reports, this finding should be interpreted cautiously, although it suggests such a possibility. The antigenicity of foods in FPIES is still a poorly understood facet of this disease [5], and further case series are needed to shed light on the mechanism involved.

The patient had typical symptoms at the age of one year, but FPIES remained undiagnosed for more than two years. Diagnosing FPIES tends to be difficult and time-consuming; a median duration of six months from symptom onset to the first consultation for wheat FPIES was reported [6]. In the present case, tolerance to common wheat products resulting in minimal dietary restriction may have contributed to the delay in diagnosis. In addition, the absence of severe symptoms and the COVID-19 pandemic may have further contributed to the delay in diagnosis. To diagnose FPIES early, it is important to appropriately assess the patient’s dietary history, consider the disease in the differential diagnosis, and perform an oral food challenge (OFC). In addition, cases that do not meet the diagnostic criteria may further delay diagnosis [10].

In the present case, remission was confirmed by a repeat OFC performed one year after diagnosis, in line with the international consensus guidelines [8]. Previous reports have suggested that wheat-induced FPIES often resolves during early childhood, although the timing varies [11]. Confirming tolerance is clinically important, particularly for staple foods such as wheat. Prolonged and unnecessary dietary elimination may negatively affect nutritional status and quality of life [5]. Therefore, follow-up and repeat OFC should be considered to assess tolerance acquisition and minimize avoidable dietary restrictions.

## Conclusions

This case suggests that variability in clinical reactivity to different wheat-based products may occur in FPIES. Careful dietary history may justify targeted OFC using specific wheat sources to establish an accurate diagnosis and avoid unnecessary dietary elimination. Given the limited available data, further studies are needed to better understand the role of wheat composition in FPIES.
